# New species of *Retiboletus* (Boletales, Boletaceae) from China based on morphological and molecular data

**DOI:** 10.3897/mycokeys.67.51020

**Published:** 2020-05-14

**Authors:** Hai-Ying Liu, Yan-Chun Li, Tolgor Bau

**Affiliations:** 1 Institute of Mycology, Jilin Agricultural University, Changchun 130118, China Jilin Agricultural University Changchun China; 2 Key Laboratory for Plant Diversity and Biogeography of East Asia, Kunming Institute of Botany, Chinese Academy of Sciences, 650201, Kunming, China Kunming Institute of Botany, Chinese Academy of Sciences Kunming China

**Keywords:** Boletes, morphology, new taxa, phylogeny, taxonomy

## Abstract

Species of the genus *Retiboletus* in China were investigated based on morphology and phylogenetic analysis of DNA sequences from the nuclear ribosomal large subunit (nrLSU) and the translation elongation factor 1-α gene (TEF1-α). Nine species were recovered from China, including two new and seven known species. The new species, namely *Retiboletus ater* and *R. sinogriseus*, from southwestern and northeastern China respectively, are documented and illustrated in this paper. *Retiboletus ater* is morphologically characterized by its black to grayish black pileus, white to grayish hymenophore, black to blackish stipe and white to grayish white context. *Retiboletus sinogriseus* is morphologically characterized by its brown to grayish-brown pileus, yellow to grayish-yellow hymenophore, pale yellow to brownish stipe and yellow to brownish-yellow context. Descriptions and line drawings of these two novel species and their comparisons with allied taxa are presented.

## Introduction

The genus *Retiboletus* Manfr. Binder & Bresinsky was erected to accommodate *Boletus ornatipes* Peck and allied species (Binder and Bresinsky 2002). The genus is morphologically characterized by the combination of the following characters: pileus convex to plane, dry, subtomentose, black, dark gray, mustard yellow or olive-brown; hymenophore pallid, grayish or yellow, unchanging or staining brown or orange-brown when bruised; hymenial cystidia present; stipe reticulate; context pallid, yellow or vivid yellow, unchanging or bruising orange-brown; clamp connections absent; spore deposit olive-brown to yellow-brown, basidiospores smooth, ellipsoid to subfusoid, inamyloid and partly dextrinoid (Binder and Bresinsky 2002; Zeng et al. 2016). The separation of *Retiboletus* from *Boletus* s. str. and its establishment at the generic rank is strongly supported (Wu et al. 2016; Zeng et al. 2016, 2018; Badou et al. 2018). So far thirteen species of this genus have been described from North/Central America and East Asia, seven out of which have been reported from China (Binder and Bresinsky 2002; Wu et al. 2016; Zeng et al. 2016, 2018).

During field investigation of Boletaceae across China, we encountered two impressive *Retiboletus* species from southwestern and northeastern China, respectively. These species can be easily recognized by their conspicuous colors in the field. Molecular phylogenetic analysis of this genus based on the nuclear ribosomal large subunit (nrLSU) and the translation elongation factor 1-α gene (TEF1-α) indicated that they represent two distinct species. Combined with morphological characters, *Retiboletus ater* and *R. sinogriseus*, are proposed and described herein. It is noteworthy that an additional collection from northeastern China, labeled R. aff. kauffmanii (HY56), was included in our molecular phylogenetic analysis. But its classification can’t be clarified due to its paucity of mature material. Further collections are needed to better estimate its taxonomic status.

## Materials and methods

### Morphological studies

Specimens were described and photographed in the field and deposited in the Herbarium of Kunming Institute of Botany, Chinese Academy of Sciences (KUN) and Herbarium of Jilin Agriculture University (HMJAU). In the descriptions, macroscopic characters were based on field notes and color slides of the specimens. Color codes are from Kornerup and Wanscher (1981). Microscopic characters were from the observations of the specimens through light microscopy. For microscopic study, dried materials were sectioned and mounted in 5% KOH solution. Sections of the pileipellis were cut tangentially and halfway between the center and margin of the pileus. All measurements were made in KOH mounts and observed under the light phase. All line drawings of microstructures were made from rehydrated material. Melzer’s reagent was used for testing color reactions of the tissue fragments to the solution. The notations “basidiospores (n/m/p)” indicate that the measurements were made on n basidiospores from m basidiomata of p collections. The expressions (a)b–c(d) stand for the dimensions of basidiospores; the range b–c contains a minimum of 90% of the measured values, a and d in the brackets stand for the extreme values. The following abbreviations are used: Q (length/width ration of basidiospores) and Q_m_ (average Q ± standard deviation).

### DNA extraction, PCR and DNA sequencing

Protocols for DNA extraction, PCR, sequencing and sequence alignment followed those in Vadthanarat et al. (2019), Gelardi et al. (2019), Zhang et al. (2019) and references therein. The primer pair used for amplifying the nrLSU region was LROR and LR5 (Vilgalys and Hester 1990). DNA sequences were compiled with SeqMan (DNASTAR Lasergene 9). Sequences were aligned with MUSCLE 3.6 (Edgar 2004) and manually adjusted where necessary. Edited sequences were deposited in GenBank (Table 1).

### Phylogenetic analysis

19 sequences (10 of nrLSU and 9 of TEF1-α) from 10 collections were newly generated in this study and aligned with selected sequences from GenBank and previous studies (Binder and Bresinsky 2002; Ortiz-Santana et al. 2007; Zeng et al. 2016, 2018) (Table 1). *Boletus edulis* Bull. and *Boletus reticuloceps* (M. Zang, M.S. Yuan & M.Q. Gong) Q.B. Wang & Y.J. Yao were chosen as outgroup. The combined nuclear dataset (nrLSU + TEF1-α) was analyzed with maximum likelihood (ML). Maximum-likelihood tree generation and bootstrap analysis were performed with the program RAxML 7.2.6 (Stamatakis 2006) running 1000 bootstrap replicates combined with a ML search.

**Table 1.** Specimens used in molecular phylogenetic study and their GenBank accession numbers.

Species	Voucher	Locality	Accession		Reference
			nrLSU	TEF1-α	
*Retiboletus ater*	Li1215	SW China	**MT010611**	**MT010621**	**This study**
*R. ater*	Li1224	SW China	**MT010612**	**MT010622**	**This study**
*R. brunneolus*	LC_LJW237	SW China	**MT010615**	**MT010625**	**This study**
*R. brunneolus*	Li993	SE China	KF112424	KF112179	Wu et al. 2016
*R. flavoniger*	RH7247	Costa Rica	AF456828	–	Binder and Bresinsky 2002
*R. flavoniger*	RH7189	Costa Rica	AF456829	–	Binder and Bresinsky 2002
*R. fuscus*	Wu445	SW China	KT990636	KT990830	Wu et al. 2016
*R. fuscus*	Cui47	SW China	**MT010614**	**MT010624**	**This study**
*R. griseus*	BD210	USA	HQ161858	–	Binder and Bresinsky 2002
*R. griseus*	snBoth	USA	KF030308	KF030414	Binder and Bresinsky 2002
*R. griseus*	Halling10162	USA	**MT010608**	**MT010618**	**This study**
*R. kauffmanii*	Wu317	SW China	KP739282	KP739301	Zeng et al. 2016
*R. nigerrimus*	Tyni1	Japan	AF456832	–	Binder and Bresinsky 2002
*R. nigrogriseus*	FHMU2045	Southern China	MH367475	MH367487	Zeng et al. 2018
*R. nigrogriseus*	FHMU2800	Southern China	MH367476	MH367488	Zeng et al. 2018
*R. ornatipes*	201/97	USA	AF456815	–	Binder and Bresinsky 2002
*R. ornatipes*	Halling10163	USA	**MT010617**	**MT010626**	**This study**
*R. ornatipes*	161/97	USA	AF456817	–	Binder and Bresinsky 2002
*R. pseudogriseus*	Zeng647	Southern China	**MT010613**	**MT010623**	**This study**
*R. pseudogriseus*	FHMU375	Southern China	MH367477	MH367489	Zeng et al. 2018
*R. pseudogriseus*	Zeng668	SE China	KP739285	–	Zeng et al. 2016
*R. retipes*	96/97	USA	AF456830	–	Binder and Bresinsky 2002
*R. retipes*	22/97	USA	AF456831	–	Binder and Bresinsky 2002
*R. retipes*	116/96	USA	AF456823	–	Binder and Bresinsky 2002
*R. retipes*	57/97	USA	AF456811	–	Binder and Bresinsky 2002
*R. sinensis*	Zeng1299	SE China	KP739291	KP739303	Zeng et al. 2016
*R. sinensis*	Zeng1278	SE China	KP739289	KP739302	Zeng et al. 2016
*R. sinensis*	Zeng569	Southern China	KP739286	–	Zeng et al. 2016
*R. sinogriseus*	LJ258	NE China	**MT010610**	**MT010620**	**This study**
*R. sinogriseus*	LJ260	NE China	**MT010609**	**MT010619**	**This study**
*R.* sp.	CAL_F_1397	India	KY290586	–	GenBank
R. aff. kauffmanii	HY56	NE China	**MT010616**	–	**This study**
*R. vinaceipes*	CFMR:DR-1035	Dominican Republic	MN250180	–	Ortiz-Santana et al. 2007
*R. vinaceipes*	CFMR:BZ-2386	Belize	MN250190	–	Ortiz-Santana et al. 2007
*R. zhangfeii*	Li1951	SE China	JQ928627	JQ928582	Wu et al. 2016
*R. zhangfeii*	Li1073	SE China	KT990630	KT990824	Wu et al. 2016
*Boletus edulis*	HMJAU4637	NE China	KF112455	KF112202	Wu et al. 2016
*B. reticuloceps*	Liang521	SW China	KT990537	KT990739	Wu et al. 2016
*Pseudoaustroboletus valens*	LF690	Southern China	KM274870	KM274878	Li et al. 2014
*P. valens*	Li915	SE China	KM274869	KM274877	Li et al. 2014

Sequences obtained in this study are shown in bold. SW = southwestern; NE = northeastern; SE = southeastern.

## Result

### Molecular analysis

**Figure 1.**
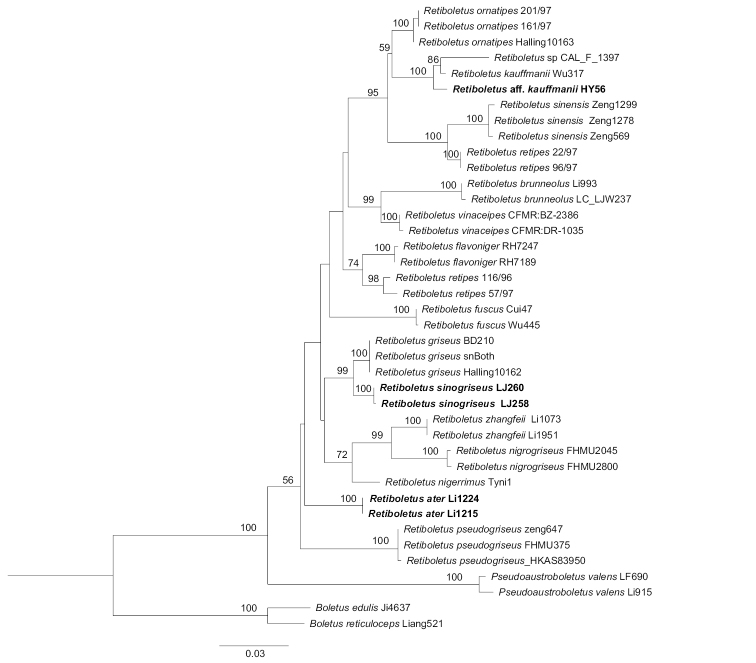
Maximum likelihood phylogenetic tree of *Retiboletus* inferred from the combined nuclear dataset (nrLSU + TEF1-α). Bootstrap frequencies (> 50%) are shown above supported branches. Newly sequenced collections are boldfaced in black. Species vouchers are provided after the species name.

The combined nuclear dataset (nrLSU + TEF1-α) consists of 64 sequences and is 1526 bp long. The alignment was submitted to TreeBASE (S25798). Phylograms with branch lengths inferred with RAxML, including the support values, are illustrated (Fig. 1). The monophyly of *Retiboletus* was moderately supported (bootstrap = 56%) in our analysis (Fig. 1). Two new species were recovered within *Retiboletus*, including two collections of *R. ater* from southwestern China and two collections of *R. sinogriseus* from northeastern China. Phylogenetically, sequences of *R. ater* form a unique lineage with 100% bootstrap support, while *R. sinogriseus* is closely related to *R. griseus* with high bootstrap support (99%). The collection labeled R. aff. kauffmanii (HY56) from northeastern China, clusters together with *R. kauffmanii* and another Indian species labeled *Retiboletus* sp. (CAL_F_1397).

### Taxonomy

#### Retiboletus ater

Taxon classificationFungiBoletalesBoletaceae

Yan C. Li & T. Bau sp. nov.

BDC8EB8A-7B6E-5C4F-914A-3DE028DCE6E9

834293

Figures 2a–c , 3

##### Etymology.

*ater* referring to the color of the basidiomata.

##### Type.

China. Yunnan Province: Jingdong County, Ailaoshan National Nature Reserve, alt. 2500 m, 14 July 2008, Y.C. Li 1215 (holotype: KUN-HKAS 56069!).

**Figure 2.**
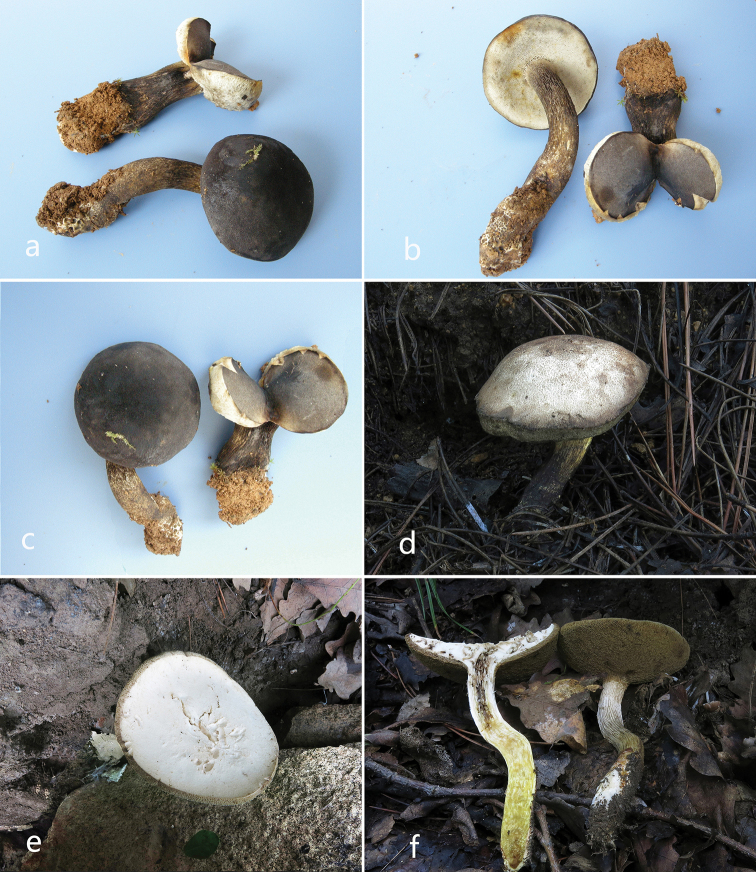
Habitat of the new *Retiboletus* species. **a–c***R. ater* (from KUN-HKAS 56069) **d–f***R. sinogriseus* (**d** from KUN-HKAS 91288 **e–f** from KUN-HKAS 91286).

##### Description.

Basidiomata small to medium-sized. Pileus 3–5 cm in diameter, hemispherical to applanate, surface dry, densely subtomentose, black (4F3) to blackish (4E2) in the center and gray (3D1) or yellowish-gray (3C2-3) towards margin, context 2.5 cm thick in the center of the pileus, pallid gray (2D1) to cream (2C3-4), unchanging when bruised. Hymenophore adnate or slightly depressed around apex of stipe; pores angular, tubes up to 11 mm long, 0.3–1 mm wide, white (2B1) when young and yellowish (2A2) in age, becoming brownish-yellow (5C7-8) when injured. Stipe 4–6 × 0.8–1.2 cm, clavate to flexuous, solid; surface dry, blackish to gray, prominently and coarsely reticulate over the upper 1/3; context white (2A1) in the upper part and yellowish to cream yellow downwards, unchanging when injured; basal mycelium white (2A1). Taste and odor indistinct.

Basidia 26–38 × 6–10 μm, clavate, thin-walled, 4-spored, hyaline to yellowish in KOH. Basidiospores [60/3/2] (7)8–10.5(11) × 3–4.5(5) μm [Q = (1.89) 2–3.33 (3.67), Q_m_ = 2.52 ± 0.42], subfusiform and inequilateral in side view with shallow suprahilar depression, elongate fusoid or narrowly oblong in ventral view, slightly thick-walled (up to 0.5 μm), brownish to yellowish-brown in KOH, olive-brown to brown in Melzer’s reagent, smooth. Hymenophoral trama boletoid; hyphae cylindrical, 3.5–9 μm wide, hyaline to yellowish in KOH, yellowish to brownish-yellow in Melzer’s reagent. Cheilo- and pleurocystidia 26–55 × 6–10 μm, abundant, subfusiform to fusiform, thin-walled, with yellowish-brown contents, surface without encrustations. Caulocystidia forming the reticulum over the stipe surface, similar to cheilo- and pleurocystidia. Pileipellis a trichoderm about 280 μm thick, composed of more or less vertically arranged, slightly interwoven, brown to dark brown hyphae, 5–15 μm wide; terminal cells 45–111 × 9–15 μm, narrowly clavate to subcylindrical or subfusiform, sometimes narrowly mucronate, rostrate, slightly thick-walled (up to 0.5 μm), hyaline to yellowish in KOH, yellowish to brownish-yellow in Melzer’s reagent. Pileal trama composed of thin- to slightly thick-walled (up to 0.5 μm) hyphae, 5–11 μm wide, hyaline to yellowish in KOH, yellowish to brownish-yellow in Melzer’s reagent. Clamp connections absent in all tissues.

**Figure 3.**
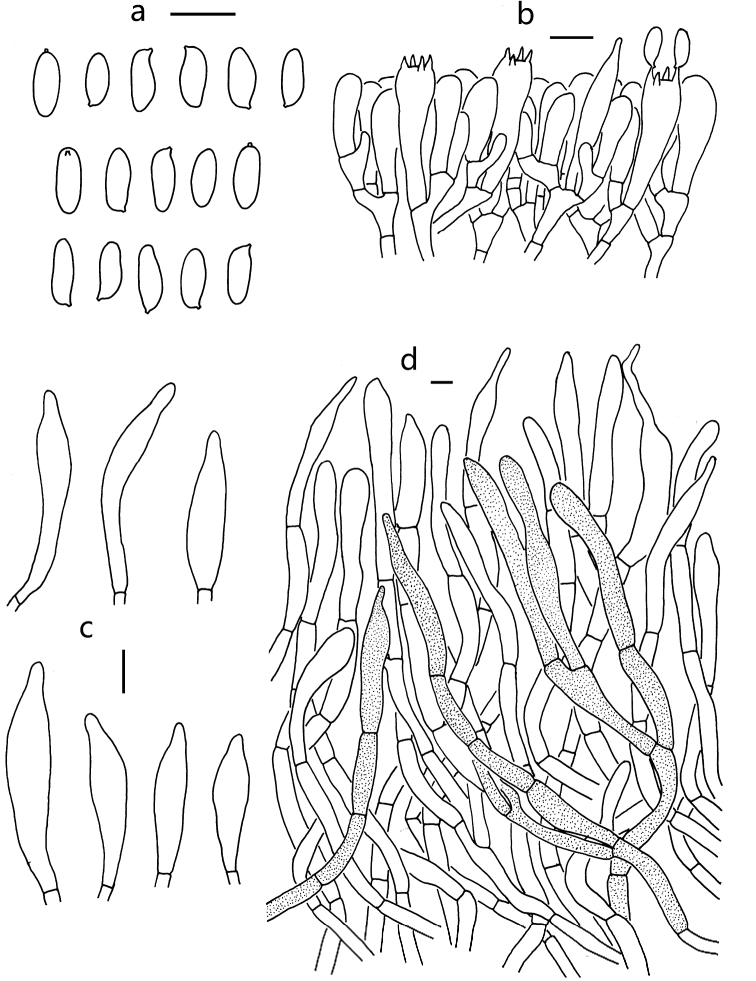
Microscopic features of *R. ater* (KUN-HKAS 56069). **a** Basidiospores **b** basidia and pleurocystidium **c** cheilo- and pleurocystidia **d** pileipellis. Scale bars: 10 μm.

##### Habitat, ecology and distribution.

Solitary on the ground in forests dominated by plants in the family Fagaceae; currently known from southwestern China.

##### Additional specimens examined.

China. Yunnan Province: Jingdong County, Ailaoshan National Nature Reserve, alt. 2500 m, 14 July 2008, Y.C. Li 1224 (KUN-HKAS 56078).

##### Discussion.

*Retiboletus ater* is characterized by the black to blackish or gray to yellowish-gray pileus, the white to yellowish hymenophore, the gray to brownish-gray stipe, the prominent and coarse reticulum over the upper 1/3 of the stipe and the trichoderm pileipellis with hyphae 9–15 μm wide. It generally shares the same colored pileus and hymenophore with *R. fuscus* (Hongo) N.K. Zeng & Zhu L. Yang, *R. griseus* (Frost) Manfr. Binder & Bresinsky, *R. nigrogriseus* N.K. Zeng, S. Jiang & Zhi Q. Liang, and *R. pseudogriseus* N.K. Zeng & Zhu L. Yang. However, *R. fuscus* is characterized by an overall reticulate stipe, slight longer basidiospores (9–12 × 3.5–4.5 μm) and narrower pileipellis hyphae (4–8 μm wide) (Zeng et al. 2016). *Retiboletus griseus* has a reticulum over the upper 2/3 of the stipe, a cream or grayish-brown stipe often with orange-yellow stains when hurt, and a distribution in North/Central America (Smith and Thiers 1971; Ortiz-Santana et al. 2007). *Retiboletus nigrogriseus* is characterized by the white to olivaceous contex in the stipe, the entirely reticulate stipe and the cutis pileipellis with hyphae 4–10 μm wide. *Retiboletus pseudogriseus* has a grayish white pileus which is covered with brown to blackish brown squamules, white context becoming brown when injured, and a slender and completely reticulate stipe.

In the phylogenetic analysis (Fig. 1), *R. ater* forms an independent lineage within *Retiboletus*, future studies would require more molecular sequence data to help fully resolve its evolutionary relationships to the other species.

#### Retiboletus sinogriseus

Taxon classificationFungiBoletalesBoletaceae

Yan C. Li & T. Bau sp. nov.

E4227468-BD4D-562F-9F6D-8E71FFF13E81

834294

Figures 2d–f , 4

##### Etymology.

*sino* (Latin) = China, reflecting that the basidiomata were collected in China + *griseus* for similarity of the basidiomata of this species to *Retiboletus griseus*.

##### Type.

China. Liaoning Province: Anshan City, Qianshan, alt. 400 m, 25 Aug 2015, J. Li 260 (holotype: KUN-HKAS 91288!).

**Figure 4.**
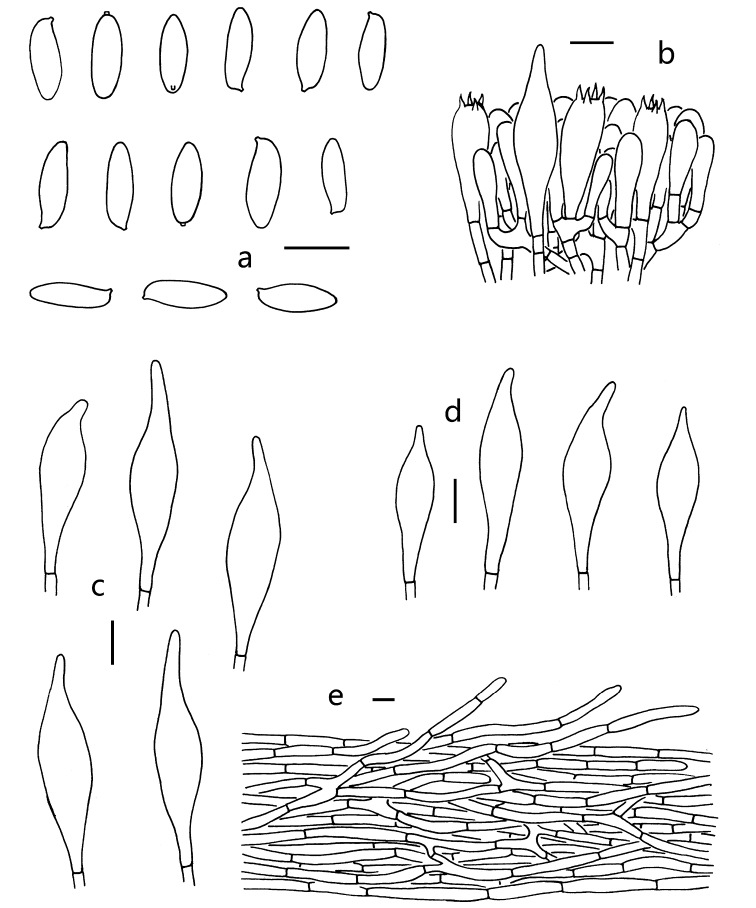
Microscopic features of *R. sinogriseus* (KUN-HKAS 912889). **a** Basidiospores **b** basidia and pleurocystidium **c** pleurocystidia **d** cheilocystidia **e** pileipellis. Scale bars: 10 μm.

##### Description.

Basidiomata medium-sized. Pileus 6–7.2 cm in diameter, subhemispherical to applanate, sometimes convex; surface tomentose, grayish-brown (5D2-3) to brown (5E4), rimose when dry, always cracked into small squamules on grayish (4B1) to whitish (2A1) background; context 1–2 cm thick in the center of pileus, white (1A1), unchanging when injured. Hymenophore adnate, sometimes slightly depressed around apex of stipe; pores angular, 0.3–1 mm wide, tubes up to 14 mm long, yellow (4C3-4) to grayish-yellow (4B2-3), unchanging when injured. Stipe 6–8 × 1.1–1.5 cm, subcylindrical, solid; surface dry, pale yellow at apex, blackish-yellow towards the base, entirely covered with moderately developed reticulum; context white to cream in the upper part and yellowish to yellow downwards, unchanging when injured; basal mycelium yellow. Taste and odor indistinct.

Basidia 21–27 × 9–11 μm, clavate, thin-walled, four-spored; sterigmata 4–5 μm long. Basidiospores [40/2/2] (9) 10.0–13.5 (–14.0) × (3) 4.0–5.0 (–5.5) μm, Q = (2.25–) 2.5–3.25 (–3.42), Q_m_ = 2.88 ± 0.32, subfusiform to ellipsoid, slightly thick-walled (up to 0.5 μm), hyaline to yellowish in KOH, olive-brown to yellowish-brown in Melzer’s reagent, smooth. Hymenophoral trama boletoid. Cheilo- and pleurocystidia 35–56 × 7–12 μm, abundant, subfusiform to fusiform, thin-walled, with yellowish-brown to brown contents, without encrustations. Pileipellis a subcutis, 100–120 μm thick, composed of thin-walled filamentous hyphae 4–7 μm wide, with subcylindrical to clavate terminal cells 33–72 × 4–6 μm, sometimes with subacute apex, colorless to pale yellowish-brown in KOH, yellow-brown to brownish in Melzer’s reagent. Pileal trama composed of thin-walled hyphae 4–9 μm wide, colorless to pale yellowish-brown in KOH, yellow-brown to brownish in Melzer’s reagent. Clamp connections absent in all tissues.

##### Habitat, ecology and distribution.

Solitary on the ground in mixed forests dominated by plants in the families Fagaceae and Pinaceae; currently known from northeastern China.

##### Additional specimens examined.

China. Liaoning Province: Anshan City, Qianshan, alt. 400 m, 25 Aug 2015, J. Li 258 (KUN-HKAS 91286).

##### Discussion.

*Retiboletus sinogriseus* has a grayish-brown to brown pileus, a pale yellow to blackish-yellow stipe. Such traits are very similar to those of *R. griseus*. Interestingly, *R. sinogriseus* clusters with *R. griseus* with strong statistical support (Fig. 1). However, *R. griseus*, originally described from North America but not found in China yet, has a distinctly pallid hymenohore and broad pileipellis hyphae which are up to 17 μm wide (Singer 1947; Smith and Thiers 1971; Ortiz-Santana et al. 2007). Additionally, the *R. sinogriseus*/*R. griseus* clade is clustered with *R. zhangfeii* N.K. Zeng & Zhu L. Yang, *R. nigrogriseus* and *R. nigerrimus* (R. Heim) Manfr. Binder & Bresinsky (however without bootstrap support). In this assemblage, *R. zhangfeii* differs significantly from *R. sinogriseus* by its differently colored pileus, hymenophore, stipe and context (Zeng et al. 2016). *Retiboletus nigrogriseus* has a black to gray pileus, white to grayish white hymenophore, white to olivaceous contex in the stipe and much smaller basidiospores 8–10.5 × 3.5–4.5 μm. *Retiboletus nigerrimus*, originally described from Papua New Guinea, has a pileus with a distinctive blue tinge, a context lemon yellow in pileus and orange in the base of stipe and longer and narrower basidiospores 11.5–14.5 × 3.6–4.6 μm (Heim 1963).

Nine species of *Retiboletus* were recorded from China, including two new species described herein. For the convenience of identification, a key to the species in China is given below.

### Key to *Retiboletus* species in China

**Table:** 

1	Hymenophore bright yellow to brownish-yellow, stipe yellow to orange-yellow, mycelium on the base of stipe yellow to brownish-yellow	**2**
–	Hymenophore whitish to grayish white, stipe black to blackish or grayish-black, mycelium on the base of stipe whitish to grayish-white	**4**
2	Pileus grayish-brown to brown without olivaceous tinge, context in pileus white to grayish-white	***R. sinogriseus***
–	Pileus yellow-brown to olive-brown, context in pileus yellow to pale yellow	**3**
3	Basidiomata medium-sized to large, pileus up to 15 cm in diameter, basidiospores 9–13 × 4–5 μm, cheilo- and pleurocystidia 30–60 × 6–10 μm	***R. kauffmanii***
–	Basidiomata small to medium-sized, pileus up to 8 cm in diameter, basidiospores 8–11 × 3.5–4 μm, cheilo- and pleurocystidia 20–46 × 4.5–7 μm	***R. sinensis***
4	Context in the stipe white to grayish-white with olivaceous tinge	**5**
–	Context in the stipe white to grayish-white with grayish-yellow tinge	**6**
5	Hymenophore white when young, lilac to purplish when old, basidiospores 9–11 × 4–5 μm	***R. zhangfeii***
–	Hymenophore white to grayish-white without lilac to purplish tinge, basidiospores relatively small 8–10.5 × 3.5–4 μm	***R. nigrogriseus***
6	Stipe entirely reticulate	**7**
–	Stipe without reticulum or with reticulum restricted to the upper part	**8**
7	Pileus brown to blackish-brown, basidiospores 9.5–11 × 4–4.5 μm, pileipellis hyphae up to 8 μm wid	***R. pseudogriseus***
–	Pileus grayish-brown to grayish-black, basidiospores slightly narrower 9–12 × 3.5–4 μm, pileipellis hyphae broad up to 13 μm wide	***R. fuscus***
8	Pileus pale brown to grayish-brown, stipe without reticulum, basidiospores 10–12.5 × 4.5–5 μm, pleurocystidia 50–80 × 9–14.5 μm	***R. brunneolus***
–	Pileus black to blackish, stipe covered with reticulum over the upper 1/3, basidiospores much smaller 8–10.5 × 3–4.5 μm, pleurocystidia relatively small 26–55 × 6–10 μm	***R. ater***

## Supplementary Material

XML Treatment for Retiboletus ater

XML Treatment for Retiboletus sinogriseus
